# Supplementary material for: Semaglutide Enhances Cellular Regeneration in Skin and Retinal Cells In Vitro
Source: Pharmaceutics. 2025 Aug 27;17(9):1115. doi: 10.3390/pharmaceutics17091115 (PMC12473120; doi:10.3390/pharmaceutics17091115)
Supplement: Supplementary file 1 [file pharmaceutics-17-01115-s001.zip › pharmaceutics-3701002-supplementary-revised.pdf]

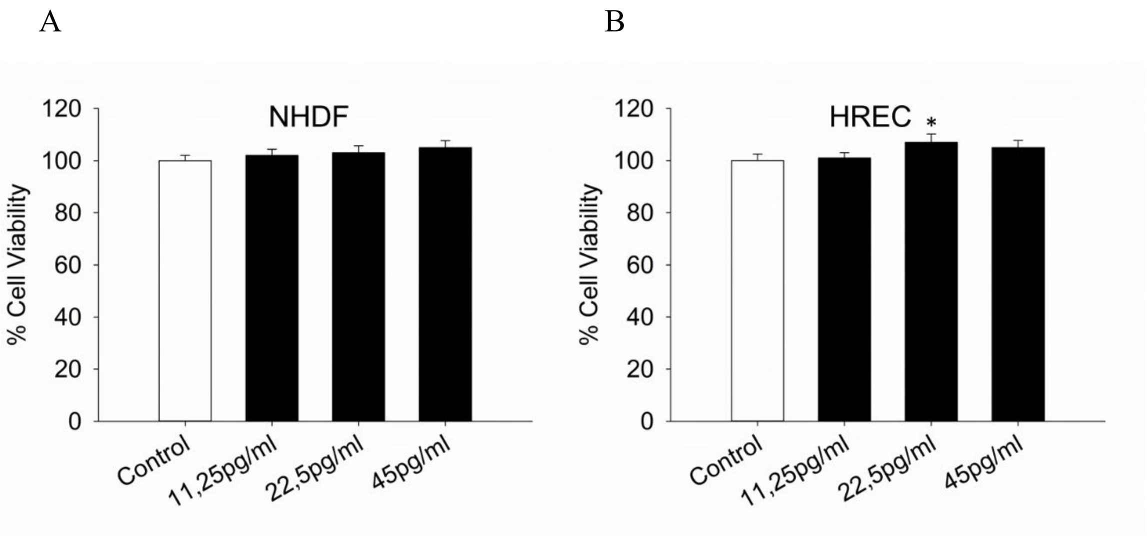

Supplementary Figure S1: Assessment of cell viability

A, B. Cell viability levels of NHDF and HREC were assessed using the MTT assay. Cells were cultured with varying concentrations of semaglutide (control: medium only without H_2O_2); 11,25, 25,5, and 45 pg/ml) for 24 hours. The results are expressed as mean ± SD from three independent experiments (N=3). Statistical analysis was performed using one-way ANOVA followed by Dunnett's T3 post-hoc test. Significance levels are indicated as $^*P < 0.05$, $^{**}P < 0.01$, and $^{***}P < 0.001$ when compared to the control group.

ANOVA, analysis of variance; HREC, human retinal endothelial cells; MTT, 3-(4,5-dimethylthiazol-2-yl)-2,5-diphenyltetrazolium bromide; NHDF, normal human dermal fibroblasts; SD, standard deviation

A B

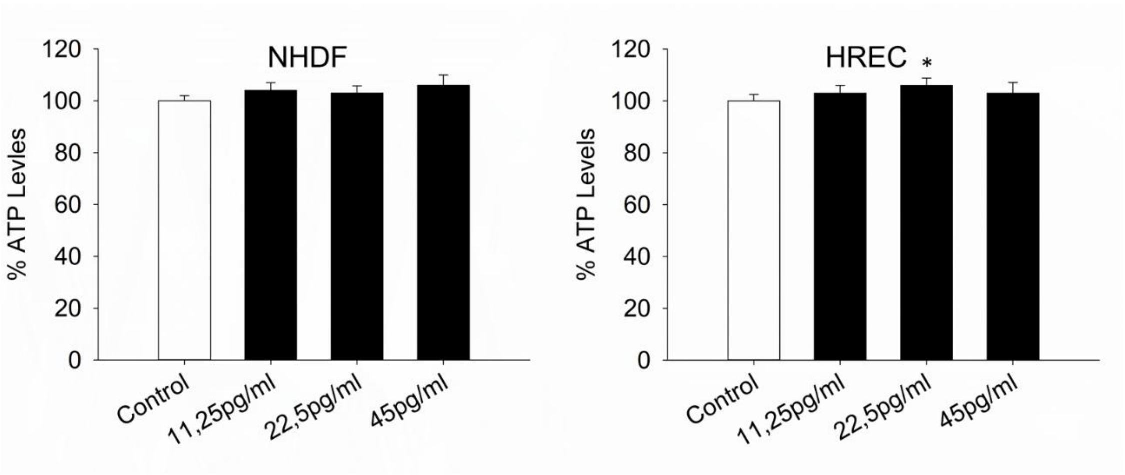

Supplementary Figure S2: Measurement of ATP levels in NHDF and HREC

A, B. ATP levels as determined by Vialight Plus assay kit. NHDF and HREC cultured with semaglutide at various concentrations (control: medium only without H_2O_2); 11,25, 25,5, and 45 pg/ml) for 24 hours. The data are expressed as mean ± SD of three independent experiments (N=3). Statistical significance was determined by one-way ANOVA followed by the Dunnett's T3 post-hoc test; where *P-value <0.05; **P-value <0.01; ***P-value <0.001 in comparison with control group.

ANOVA, analysis of variance; ATP, adenosine triphosphate; HREC, human retinal endothelial cells; NHDF, normal human dermal fibroblasts; SD, standard deviation.

Supplemental Table S1: Gene name. Accesion No. Primer sequences, Anealinh temperature					
Gene Symbol	Gene Name	Accession No	Primer F (5'-3')	Primer R (5'-3')	Annealing Temperature (ºC)
ACTB	actin. beta	NM_001101.5	GAGAAGAGCTACGAGCTGCC	AAGGAAGGCTGGAAGAGTGC	60
COL1A1	collagen. type I. alpha 1	NM_000088.4	AGTGGTTTGGATGGTGCCAA	GCACCATCATTTCCACGAGC	60
COL3A1	collagen. type III alpha 1	NM_000090.4	AGCCTGGTAAGAATGGTGCC	CTCCTGGGATGCCATTTGGT	60
COL4A1	collagen. type IV alpha 1	NM_001303110.2	AGGAGTGCCATTGCTTTTTCAA	TGGAAACCAGTCCATGCTCG	60
COL6A1	collagen. type VI alpha 1	NM_001848.3	TTATCAACGACGCCACCGA	TGCGAGTTGCCATCTGAGAAG	60
GPX1	glutathione peroxidase 1	NM_001329455.2	CGATGTTGCCTGGAACTTTGAG	ATGTCAATGGTCTGGAAGCGG	60
GPX4	glutathione peroxidase 4	NM_001845.6	GGAGTTTAGAAGTGCGCCATTC	CCAAAAGCTGTAAGCGTTTGC	60
CAT	catalase	NM_001752.4	ACCAAGGTTTGGCCTCACAA	CCACCCTGATTGTCCTGCAT	60
SOD1	superoxide dismutase 1	NM_000454.5	GGATGAAGAGAGGCATGTTGGA	TAGACACATCGGCCACACCAT	60
Il1B	interleukin 1 beta	NM_000576.3	TTTGAGTCTGCCCAGTTCCC	TTTGGTCCCTCCCAGGAAGA	60
IL2	Interleukin 2 (IL2)	NM_000586.4	GACCCAGGGACTTAATCAGCAAT	GCTGTCTCATCAGCATATTCACAC	60
MMP9	matrix metallopeptidase 9 (MMP9)	NM_004994.3	GTACTCGACCTGTACCAGCG	AGAAGCCCCACTTCTTGTCG	60
MMP3	matrix metallopeptidase 3 (MMP3)	NM_002422.5	ACTCCCTGGGTCTCTTTCACT	GAACCGAGTCAG	60

				GTCTGTGAG	
EGF	Epidermal growth factor (EGF)	NM_001178130.3	AACTTCATGGAGGCAGGAGC	CATTACCTGGGGACAGGAGC	60
FGF	fibroblast growth factor (FGF)	NM_001354953.2	TTACCACGCCTTGACCTTCC	ATGGTATCCCCTCAGCCAGT	60
